# A case report: insights into reducing plastic waste in a microbiology laboratory

**DOI:** 10.1099/acmi.0.000173

**Published:** 2020-10-14

**Authors:** Joana Alves, Fiona A. Sargison, Hanne Stawarz, Willow B. Fox, Samuel G. Huete, Amany Hassan, Brian McTeir, Amy C. Pickering

**Affiliations:** ^1^​ Roslin Institute, School of Medicine and Veterinary Medicine, University of Edinburgh, Easter Bush Campus, Easter Bush, Edinburgh EH25 9RG, Scotland, UK; ^2^​ Department of Animal Medicine, Faculty of Veterinary Medicine, University of Alexandria, Egypt

**Keywords:** sustainability, plastic waste, microbiology laboratory, decontamination station

## Abstract

Single-use plastics have often replaced more sustainable materials in microbiology laboratories. Keeping in mind that one of the objectives of the United Nations Sustainable Development Goals is responsible consumption and production, we wanted to document how many single-use plastic items could be saved by taking reduction and reuse approaches in a microbiology laboratory. After taking 4 weeks to document the baseline levels of single-use plastic waste being generated in our laboratory and identifying ways to reduce our reliance on them, we implemented various reduction and reuse approaches and then documented our plastic use over a 7-week period. Reduction approaches included moving to sustainable materials, such as reusable wooden sticks for patch plating and metal loops for inoculation. Reuse approaches focused on reusing plastic tubes via a chemical decontamination station and autoclaving, facilitating the reduction of single-use plastics and a decrease in the amount of waste generated. By utilizing reduction and reuse strategies, which could be implemented in other microbiology laboratories, substantial single-use plastic savings were achieved. These savings had an impact on the amount of biohazard waste being autoclaved and incinerated, as well as generating substantial cost savings for the research institute. The reductions in waste documented in this study could act as a benchmark for others wanting to implement the changes described.

**Impact Statement**

Increasingly, the scientific community is discussing how researchers can develop more sustainable practices. Magazine articles have emphasized the essential role of science in achieving the United Nations Sustainable Development Goals and guidance has been provided by some members of the scientific community as to how to reduce research-­related plastic waste. Here we provide a case report documenting, for the first time, the plastic use of a standard microbiology laboratory and the impact of reduction and reuse approaches on the number of plastic items used, as well as the weight of waste generated. This study highlights the large reductions in single-use plastic that can be achieved in research laboratories when a sustainable mindset is applied. Through the details provided in this case report, other research laboratories will be able to adopt the same or similar approaches in an effort to reduce single-use plastic consumption in science. A widespread adoption of such approaches could have significant impacts on the levels of plastic waste generated globally and allow scientific research to become a more responsible consumer of plastic.

## Introduction

Public concern surrounding the levels of single-use plastic in our day-to-day lives has increased greatly since 2017, when the impact of plastic pollution in the sea was highlighted by the television series *Blue Planet II* and China limited the types of plastic being recycled from Europe and the USA [1]. In response, many governments have implemented changes to reduce the amount of single-use plastics, with the European Parliament banning certain plastics by 2021, such as straws, stirrers, cutlery and cotton buds [2]. Reducing single-use plastics is something that many researchers feel strongly about, and yet in the typical microbiology laboratory we are surrounded by disposable plastic, which is often not recycled due to biological contamination. Fortunately, there have been several innovators in this field. A researcher from the University of York, David Kuntin, pioneered the concept of a ‘decontamination station’ – a container allowing over 16 h soaking of plastic items in a high-level disinfectant followed by a water rinse for chemical decontamination – as a means for recycling tissue culture flasks [3]. Additionally, Tim Calder, at the University of Edinburgh, developed a glove recycling scheme in the School of Chemistry that allowed more than 1 million plastic gloves to be recycled in 2019 [4]. Such innovations are paving the way for universities to recycle single-use plastics from research laboratories.

Such recycling schemes are important but do not reduce the amount of single-use plastic consumed by research laboratories. This is particularly concerning as it has been estimated that in 2014, 5.5 million tonnes of plastic waste were generated in research laboratories worldwide, the equivalent of 83 % of all plastic recycled in 2012 [5]. Considering the United Nations Sustainable Development goal of responsible consumption and production, it is the responsibility of all scientists, including microbiologists, to consider where reductions in single-use plastic can be introduced in research laboratories [6]. Certain institutions are rising to this challenge, with the University of Leeds boldly pledging to phase out single-use plastic from the whole university, including research laboratories, by 2023 [7]. At the University of Manchester, a similar scheme has saved more than 24 000 pieces of plastic each academic year by focusing on reducing plastic in laboratory practical classes [8]. As the gold standard of the plastic waste hierarchy is to reduce rather than reuse or recycle plastics, the aim of this study was to document reductions in single-use plastics in a microbiology laboratory after the implementation of several simple reduction and reuse strategies.

## Case report

### Laboratory set-up and current sustainable practices

Similar to other microbiology laboratories, our research covers a diverse range of disciplines, from molecular biology to immunology. Therefore, the equipment and consumable needs of each researcher can vary depending on the sterility and non-pyrogenic requirements. Overall, this means that our consumable needs, especially in terms of laboratory plastics, are quite representative of the needs of other laboratory disciplines. Despite this, we are aware that there are items on our consumables list, such as inoculation loops and Petri dishes for agar media, that are specific to microbiology research.

At the Roslin Institute, University of Edinburgh, many plastic- and polystyrene-reducing measures have already been implemented [9]. For instance, ordering is centralized to promote bulk ordering with fewer shipments and reduced levels of packaging. Recycling stations are also present in the research laboratories to allow recycling of packaging as well as uncontaminated plastic bottles and tip boxes. However, these measures do not reduce the levels of single-use plastic, such as tubes, cell culture flasks, plastic loops and Petri dishes, being consumed for research purposes. Currently, at the University of Edinburgh, contaminated plastic waste is processed alongside other non-plastic biohazard waste and autoclaved to be rendered safe, making it unsuitable for recycling. Bearing in mind the large quantities of the plastic directed to the biohazard waste, we decided to focus our efforts on reducing the plastic going through this waste route.

### Evaluating our baseline plastic consumption

To quantify the effects of new sustainability guidelines on our laboratory we started by monitoring its plastic use and waste. To establish a baseline, all plastic consumable items already in use were removed from the laboratory and tissue culture room and all waste bags were emptied. A digital weight scale was introduced close to the waste bin collection point, to allow all waste bags to be measured before disposal, and three white boards were introduced into the laboratory to allow all laboratory members to note the number of plastic items collected and used from the communal stock room. After every week, the values registered on the white boards were transferred to an excel spreadsheet and the white boards were cleaned to allow weekly measurements of plastic items collected.

At the time, our research group was composed of two postdoctoral researchers, four PhD students and one master’s student working full time. The research projects involved activities related to bacterial isolation and cloning, protein purification and culture of primary cells and cell lines, all at biosafety category level 2. After four consecutive weeks, we had disposed of 97 kg of waste in biohazard waste bags and collected for use almost 2000 units of single-use microbiology plastic (loops and spreaders) and 2200 tubes (Falcon and universal tubes).

### Action plan and behaviour change

After an initial assessment of the major laboratory plastic items used in our research, we were able to identify practices that we believed could be replaced with more sustainable approaches without compromising work quality. We looked for guidance from the Roslin Institute Facilities team and recommendation guidelines from the University of Edinburgh Department of Social Responsibility and Sustainability [10]. We selected recommendations that we believed could be implemented safely in a biosafety level 2 laboratory. For that reason, we deliberately avoided the implementation of any changes to the way we deal with personal protective equipment, specifically nitrile gloves.

**Fig. 1.**
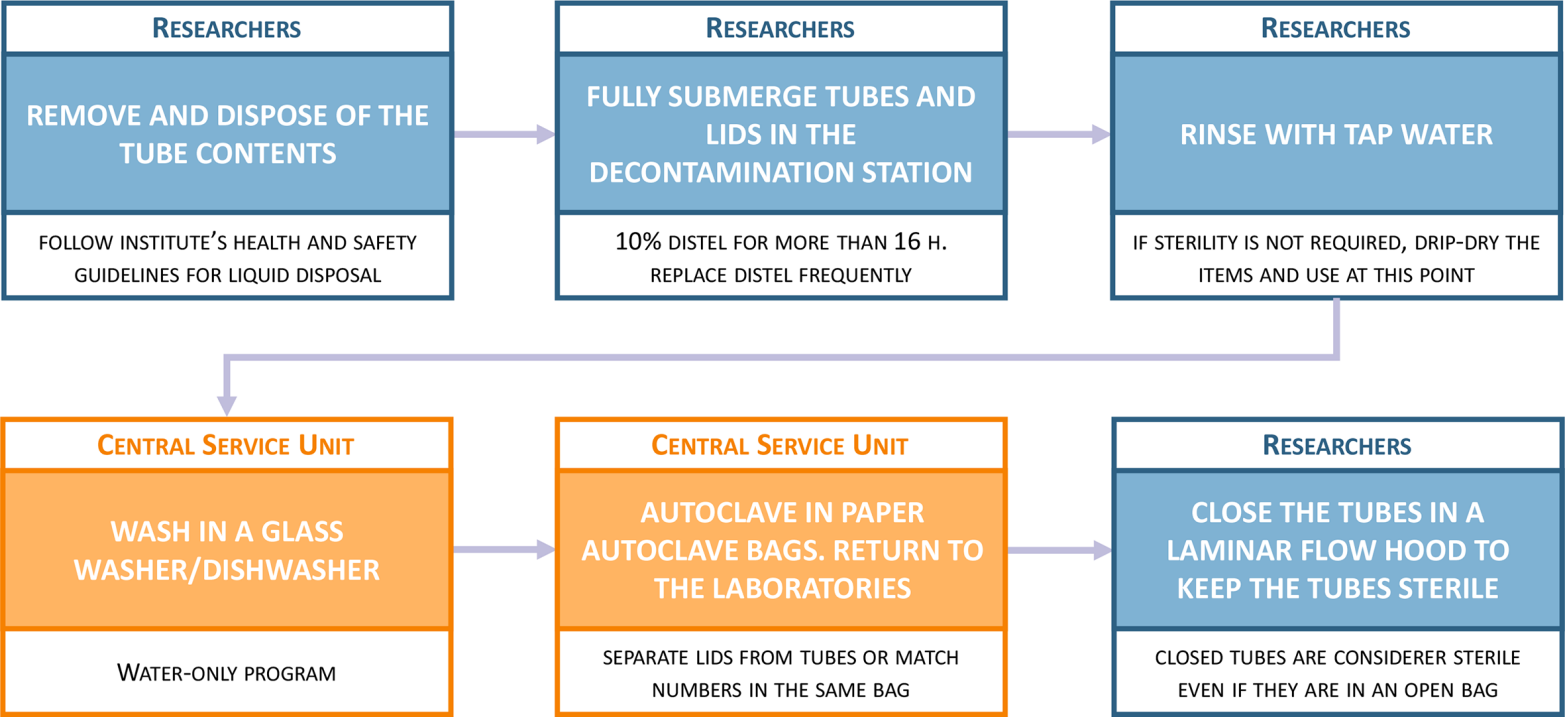
Pipeline to decontaminate and sterilize plastic tubes for reuse. Steps carried out by members of the research group are shown in blue and steps carried out by members of the central services team are shown in orange. Distel refers to Distel high level laboratory disinfectant (Scientific Lab Supplies). This workflow would be started three times a week to allow the processing of all the plastic being used in the laboratory.

As in most laboratories, a large proportion of our plastic use is with tubes, from PCR tubes for small reactions to 50 ml Falcons. We already used glass containers to grow some of our bacterial cultures, but even in these cases, plastic tubes were often used for centrifugation or additional processing. Moreover, tissue culture protocols are required to be performed in a bacterial contaminant-free environment (non-pyrogenic conditions) that, so far, can only be achieved by using non-pyrogenic single-use plastic. Nevertheless, for most microbiology protocols, this condition does not need to be met, allowing for use of autoclave-sterile tubes. For that reason, and with the help of the institute’s central services team, we established a workflow to decontaminate and autoclave plastic tubes for reuse (Fig. 1). The universal tubes available at the Roslin Institute are made from non-autoclavable polystyrene plastic and, for that reason, we replaced them with autoclavable 50 ml Falcon tubes wherever possible (see Table 1 for information on the consumables used in this study).

**Table 1.** Consumables used in this study

Item	Supplier	Catalogue no.	No. of items per purchase	Price (GBP)*	Autoclavable
**Falcon tubes**	Griener Bio-one	15 ml – 188 271	100	6.49	Yes
		50 ml – 227 261	20	1.39	Yes
**Universal tubes**	Mackay and Lynn	128A	400	35.68	No
**Inoculation loops**	Scientific Lab Supplies	SLS2008	1000	14.44	No
**Spreaders**	Fisher Scientific	12 322 048	500	22.17	No
**Stripettes**	Fisher Scientific	5 ml – 4051	200	10.48	No
		10 ml – 4101	200	11.69	No
		25 ml – 4251	200	20.46	No
**Weighing boats**	STARLAB	30 ml – E3300-0030	500	25.72	No
**Sterilization bags**	Westfield Medical Ltd	AUT1451	250	24.26	Yes
**Metal inoculation loops**	Fisher Scientific	Handle – 12 892 775	1	5.01	na
		Loops – 15 772 165	25	35.25	Yes
**Wooden inoculations sticks**	Sigma	Z740491	2000	79.70	Yes
**Distel**	Scientific Lab Supplies	TRI1366	5 litres	33.00	na
**U-bottom 96-well plate for serial dilutions**	Griener Bio-one	650 101	100	25.13	No

*Prices from Institute suppliers database on 2 April 2020.

na, not applicable.

**Fig. 2.**
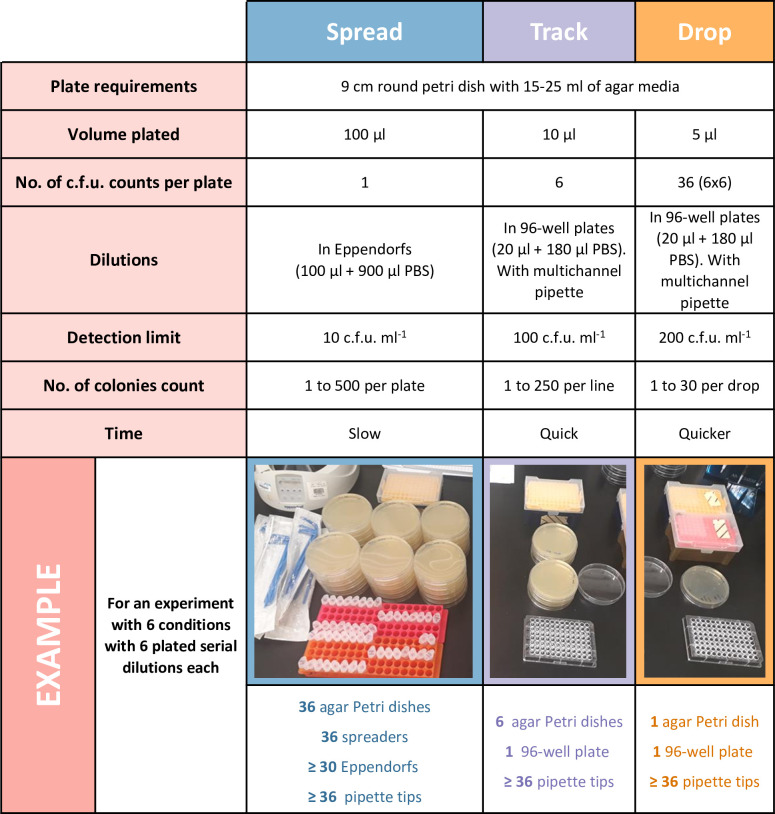
Comparison of plating techniques for colony-forming unit analysis. The more traditional spread technique is compared to track and drop techniques already described and adapted from the literature [7–10]. c.f.u., colony forming units; No., Number; PBS, phosphate buffered saline.

We also implemented guidelines for the reuse of single-use plastic items, acquired new sustainable alternatives for some items, and engaged all research group members in training and sustainability awareness sessions (Table 2). This training included the use of metal inoculation loops and reusable wooden sticks for bacterial colony picking from agar plates. We also raised awareness for the use of glass alternatives, reusable items or in-house autoclaved pipette tips when possible, instead of single-use items or filtered tips that are also available in the communal consumable store at the Roslin Institute. Bacterial culture on agar plates is one of the most common protocols performed in our laboratory, and indeed most microbiology laboratories. It is therefore a protocol responsible for a lot of our waste, especially in Petri dishes. Unfortunately, plastic Petri dishes do not keep their shape under the high autoclave temperatures, making it difficult to reuse these plastic items without creating contamination issues. Different laboratories and research teams have adopted different techniques to perform colony unit counts, with the most traditional being the spread of a single dilution onto a single agar plate. We presented our research team members with alternative techniques, such as the track [11, 12] and drop techniques [13, 14], which allow not only a considerable reduction in the materials needed, but also a reduction in the time needed to perform this protocol. The research team members performed multiple tests comparing the counts obtained with the different techniques and were happy to replace the conventional spread with the track or drop techniques (Fig. 2), depending on the experiment.

**Table 2.** Guidelines followed in this study to reduce plastic waste

Reuse of single-use items	
**Weighing boats**	Washed, dried and reused
**Plastic serological pipettes**	For pipetting common non-sterile solutions (e.g. ethanol, concentrated buffered solutions). Sheathed in their plastic wrap, labelled with the working solution that they were used for, and attached to the cupboard near the pipette controller
**Cuvettes**	Decontaminated overnight in 10 % Distel, rinsed with water, dried and reused
**Tip-collecting jars**	Contaminated tips transferred to biohazard bins when full. Chemically decontaminate the jar overnight when necessary
**Petri dishes**	Petri dishes used in cell culture room with media only are decontaminated, washed and reused for agar media with antibiotics
**Decontaminate and autoclave Falcon tubes**	
**Used 15 ml and 50 ml Falcon tubes**	Chemically decontaminated overnight, rinsed with water, washed in a dishwasher with a water-only programme, autoclaved in bags. Tubes are closed in a cell culture hood and considered sterile for non-cell culture work. We stopped using universal tubes since they cannot be autoclaved for reuse. Control*:* tested for contaminations. None so far
**Substitution of single-use items with re-usable ones**	
**Single-use**	**Replace by**
Plastic inoculation loops	Metal inoculation loops
Plastic tips for bacterial colony picking	Wooden sticks (biodegradable and reused after autoclave)
**Training**	
**Plan experiments to reduce single-use items**	Prepare master mix to reduce tips Organize experimental layouts to use the minimum number of tubes and plates Use 96-well plates instead of Eppendorf’s for serial dilutions in experiments with several dilutions/conditions Divide the agar plate in parts (more than one condition per plate) Use lines and drop technique for experiments with bacterial serial dilutions Use the same tips for the same condition
**Non-pyrogenic vs autoclaved lab plastics**	When possible use in-house autoclaved tips (with reused tip boxes and without plastic wrapping) instead of filtered tips For work that does not need to be non-pyrogenic or RNA/DNA-free, use autoclaved tubes (sterile and reused)
**Before any protocol step: is there a more sustainable way to do this?**	

Distel refers to Distel high level laboratory disinfectant (Scientific Lab Supplies).

### Lasting reductions in the number of plastic items used and the biohazard waste generated

**Fig. 3.**
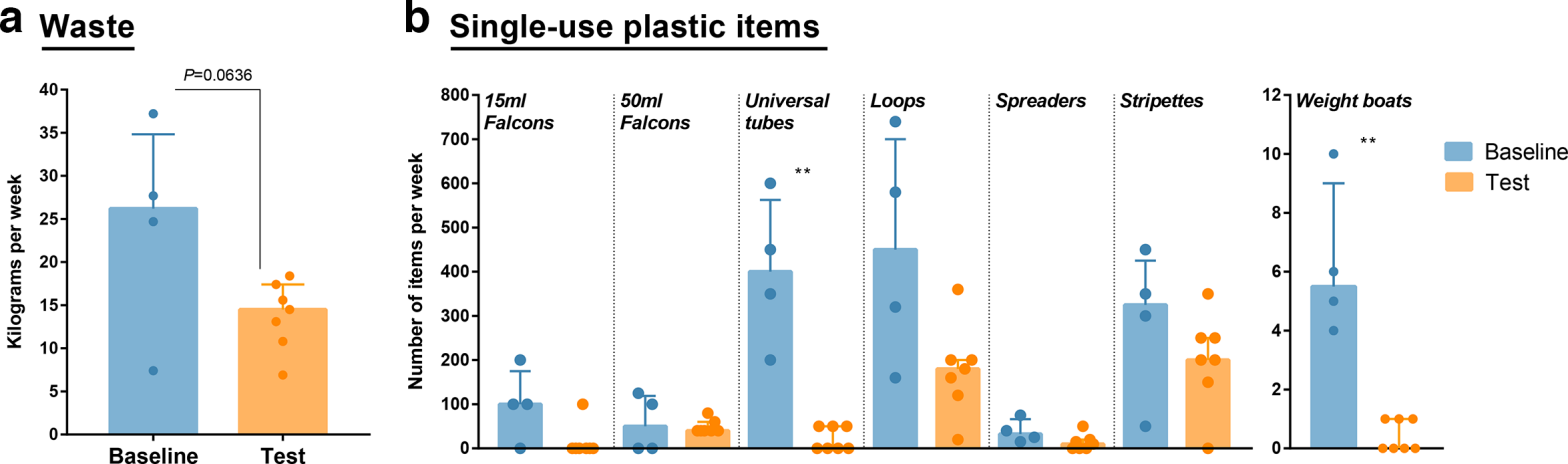
Long-lasting reductions in waste and single-use plastic items. (**a**) Weight of waste processed by the biohazard route per week. (**b**) Number of plastic items used per week. Individual week values are represented with dots and bars show median with interquartile range of baseline and test period. Data are shown for 4 weeks of the baseline measurements and 7 weeks of the test measurements. Due to large week-to-week variation, statistical differences were calculated using the non-parametric Kolmogorov–Smirnov test, **P<0.01.

To evaluate the effects of the new laboratory plastic-reducing guidelines, we measured the weight of the biohazard waste bags and monitored the use of single-use plastic items for 7 consecutive weeks after the implementation of the new measures. As expected, due to the varying work that is performed in our laboratory, variable quantities of waste and plastic items were used each week, both during the baseline and test periods (Fig. 3). Despite this high week-to-week variability, it is clear that in the 7-week period following implementation of plastic-reduction measures, we observed a reduction in the maximum waste observed (Fig. 3).

Importantly, we saw a dramatic reduction in the quantity of tubes and inoculation loops that we were using, but not in stripette use, which did not have a specific reduction strategy in place (Fig. 3B). The transition to reusable metal inoculation loops saved 1300 plastic inoculation loops over a 4-week period, the equivalent of 15 600 plastic loops per year. This saving in plastic can also be translated to a cost saving of about UK £225 per year, enough to purchase 35 metal inoculation loops with handles (see Table 1 for calculations).

When materials could not be easily replaced by more sustainable alternatives, we made efforts to reuse plastic. This was most successful with 15 and 50 ml Falcon tubes (Greiner Bio-one), which could be autoclaved for reuse. This approach has produced a dramatic reduction in the number of plastic tubes being used in our laboratory, which can be seen in Fig. 3b by the loss of universal tube use but no consequent increase in the levels of 15 or 50 ml Falcon tubes used. Over the first 4-week test period we saved approximately 1670 plastic tubes, the equivalent of 20 000 plastic tubes per year. This is a saving of UK £1390 per year, the equivalent of purchasing 467 glass culture tubes of 30 ml volume with screw-cap lids (Sigma), which can be autoclaved, allowing the replacement of plastic tubes used for culturing. Of note, we did not observe contamination related to the reuse of chemically decontaminated and autoclaved plastic tubes following the implementation of reuse guidelines, making us confident that the pipeline is appropriate for our research applications. The provision of training to our research group encouraged behavioural changes that reflected on the number of used plastic items (Fig. 3b). For instance, we observed a significant reduction in the number of plastic weighing boats (Fig. 3b). Even though this equates to a small number of weighing boats saved over a 4-week period (22 weighing boats), this significant decrease is very encouraging when considering the impact a similar behaviour change could have when implemented on a larger scale. Similarly, by reusing plastic cuvettes, which are purchased in boxes (each with 100 units), no new boxes of plastic cuvettes were purchased over a 4-month period, whereas five boxes of cuvettes (costing a total of UK £35.40) were purchased in the 4-month period preceding the test period.

Overall, these savings in terms of the number of plastic items led to a reduction of laboratory waste of 43 kg in a 4-week period (54 vs 97 kg in the 4-week baseline period) (Fig. 3a). This equates to 516 kg of waste per year that is no longer being autoclaved and then incinerated. It is important to remember that the above waste reduction measures only apply to one laboratory containing seven microbiologists. As the Roslin Institute contains roughly 200 wet-lab researchers, this could equate to more than 17 000 kg of biohazard waste saved if all laboratories take up similar plastic-reducing measures. This would be an unprecedented decrease in the waste generated at our research facility. It is the ongoing work of this project to see what systems can be implemented throughout the research institute to achieve such ambitious changes.

## Discussion

This study is unique in documenting the impact of plastic reduction and reuse approaches in a research laboratory. By replacing plastic items and creating a procedure for decontaminating plastic, we observed major reductions in the number of single-use plastic items being required for use in the laboratory and in the amount of waste being sent for decontamination and ultimately incineration. As such, this work promotes the growing interest of research facilities and academic institutions in becoming more sustainable in the future.

One such institution, University College London, has created a Laboratory Efficiency Assessment Framework (LEAF) [15] and a Sustainable-Lab Consumables Guide [16], which showcase many of the changes that research laboratories can implement in order to become more sustainable, including the practices we have implemented in this study. If these sustainable practices are to become widespread, large efforts are needed to implement sustainable training for scientists. This training would require tailored advice relevant to the research field and place of work, as not all practices will be applicable to all researchers or feasible in all locations. In some cases, significant operational and behavioural changes are needed to embed these practices into the day-to-day working of research facilities. For the current study, concerns about contamination and loss of precision had to be addressed and clear guidance was created for the research group, in addition to the implementation of contamination testing and the inclusion of media-only controls. Fortunately, as the public interest in reducing plastic waste is currently so high, now is the optimal time to be providing such training alongside implementing changes to our standard operational procedures [17].

To achieve even greater plastic reductions in our research laboratory, we are investigating the introduction of glass culture tubes, Petri dishes and stripettes to replace plastic versions. These glass items can be decontaminated by autoclaving and reused without the need for a chemical decontamination station. The reuse of plastic tips could also be implemented with machines such as those being marketed by Grenova [4]. Even with these additional measures, it is hard to imagine a research laboratory that is completely free of plastic. This is particularly the case when centrifuging samples, as glass culture tubes may not be appropriate for centrifugation, and performing tissue culture procedures that require non-pyrogenic and non-cytotoxic materials. Such conditions could not be achieved with reusable glass alternatives. In the current study, the decontaminated plastics were not used for any procedures relating to tissue culture, for the above reasons, but were instead employed for microbiological purposes. This could raise issues when introducing the described procedures to other laboratories that are largely tissue culture focused. We propose that such laboratories could feed plastic into a centralized plastic decontamination system, which allows reuse of the plastic in non-tissue culture-related research, such as microbiology.

It must also be made clear that the approaches highlighted in this study have drawbacks relating to efficiency and convenience of use. In order to avoid the use of plastic, we often reverted to more traditional microbiological techniques, such as the use of metal inoculation loops. These more traditional techniques are more time-consuming, as loops need to be decontaminated by flame and cooled between samples. This time cost, alongside the use of inoculation loops in a tissue culture hood, are the main reasons why plastic inoculation loops have not been eradicated from our laboratory (Fig. 3b). The decontamination process for plastic tubes also introduced new responsibilities for both the research team and the Roslin Institute’s central services team. Due to the amount of plastic being decontaminated, the decontamination station required emptying of plastic three times a week, which would take roughly 30 min to empty on each occasion. This requires significant dedication, by both the researchers and central services team, and may be prohibitive to implementing similar decontamination stations in other laboratories.

As a concluding remark, we would like to encourage all microbiologists, and indeed all research scientists, to assess how they can reduce the number of single-use plastic items in their day-to-day research. Events such as #LabWasteDay, and certifications from My Green Lab and LEAF, are helping to identify our plastic problem [18]. Let us start implementing the solutions.
